# The causal effects of genetically determined human blood metabolites on the risk of atrial fibrillation

**DOI:** 10.3389/fcvm.2023.1211458

**Published:** 2023-07-26

**Authors:** Tao Cheng, Huan Wang, Yuanhui Hu

**Affiliations:** ^1^ Department of Cardiological Medicine, China Academy of Chinese Medical Sciences Guang’anmen Hospital, Beijing, China; ^2^ Beijing University of Chinese Medicine, Beijing, China

**Keywords:** metabolites, atrial fibrillation, Mendelian randomization, causality, tryptophan betaine

## Abstract

**Background:**

Blood metabolites have been found related to atrial fibrillation (AF), but the causal role is still unclear. Mendel randomization (MR) can give information about the causality between blood metabolites and AF.

**Methods:**

Two-sample MR analysis was used to evaluate the causality between 486 blood metabolites and AF. Firstly, the genome-wide association study (GWAS) data for AF (from Nielsen et al.) was analyzed and some metabolites were identified. Then another GWAS data for AF (from Roselli et al.) was repeatedly analyzed to verify the results. Inverse variance weighted method was mainly used to determine the causality, and MR-egger, Weighted Median, and MR-PRESSO models were used as supplements of MR. Cochran's *Q* test was used to assess heterogeneity. And MR-Egger intercept and MR-PRESSO global test were performed to measure pleiotropy.

**Results:**

The study used Bonferroni's corrected *P* value (*P* < 1.03 × 10^−4^) as the significance threshold. After MR analysis and replication analysis, we found two overlapped metabolites. Among which tryptophan betaine was the most significant causal metabolite in both AF GWAS data (from Nielsen et al.) (odds ratio (OR) = 0.83, 95% confidence interval (CI) = 0.76–0.90, *P* = 9.37 × 10^−6^) and AF GWAS data (from Roselli et al.) (OR = 0.82, 95% CI = 0.76–0.88, *P* = 2.00 × 10^−7^), while uridine was nominally significant metabolites in both AF GWAS data (from Nielsen et al.) (OR = 0.58, 95% CI = 0.40–0.84, *P* = 0.004) and AF GWAS data (from Roselli et al.) (OR = 0.56, 95% CI = 0.35–0.88, *P* = 0.01). And the results of sensitivity analysis showed that none of them had obvious heterogeneity or pleiotropy.

**Conclusion:**

The study identified several blood metabolites that were causally related to AF, which may provide new perspectives on the pathogenesis of AF.

## 1. Introduction

Atrial fibrillation (AF) is the most common cardiac arrhythmia in clinical practice, with an incidence rate of 2.3%–3.4%, which increases with age (1). AF can increase the risk of stroke and systemic thromboembolism in patients, and the risk of heart failure increases by 1.5–2.0 times (2). It has brought a heavy burden to patients, society and economy (3). Although many studies have been done in recent years to explore the pathophysiological mechanism and treatment methods of AF, the prevention and treatment of AF is still a major problem in clinical medicine.

Metabolites are considered as the final reaction of the biological system to inherited or environmental variations, and their levels directly reflect the physiological state of the body (4). Human blood metabolites (intermediates of metabolism), vary greatly among different individuals. Cell-free metabolites (from plasma or blood) are usually used to explore their biological significance and function because of the convenience of sample collection.

Metabolomics is an important part of system biology (5). It explores biomarkers related to disease diagnosis and prognosis by detecting small molecular substances in biological samples, which has been widely used in clinical and experimental studies (6, 7).

There is now growing interest in the relationship between metabolites and AF. Many studies have found that there is a potential relationship between metabolites and AF, indicating that some metabolites take part in the pathogenesis and development of AF (8–12). Harskamp et al. found that medium chain acylcarnitines, short chain dicarboxylacylcarnitines and long chain acylcarnitines were associated with AF (8). Yan et al. found that metabolites such as potassium and sodium ion were associated with AF (9). Harju et al. found 61 metabolites associated with AF, including energy, histidine, glutathione, purine, sugar, lipid and so on (10). However, as far as we know, a comprehensive and systematic research evaluating the causal effects of blood metabolites on AF is still lacking.

Mendelian randomization (MR) is an epidemiological method that uses single nucleotide polymorphisms (SNPs) as exposure-related instrumental variables (IVs) to assess the causality between exposure and outcome (13). In the absence of randomized clinical trials (RCT), MR research is a significant alternative method to analyze the causal relationship, because MR analysis, based on the random classification theory in the process of meiosis in genetics, carried out random grouping similar to RCT in the population (14). Diseases usually occur after the formation of genotype, so genotype is difficult to be affected by diseases progression, so it is less susceptible to reverse causality (13), thus overcoming the inherent bias and confounding factors of observational studies (15, 16).

The study used the statistical data of the genome-wide association study (GWAS) as the data source and the two-sample MR analysis as the research method to evaluate the causality between blood metabolites and AF, selected the candidate blood metabolites related to AF, providing a novel perspective for the early diagnosis and treatment strategy of AF.

## 2. Methods and materials

### 2.1. Study design

**Figure 1:**
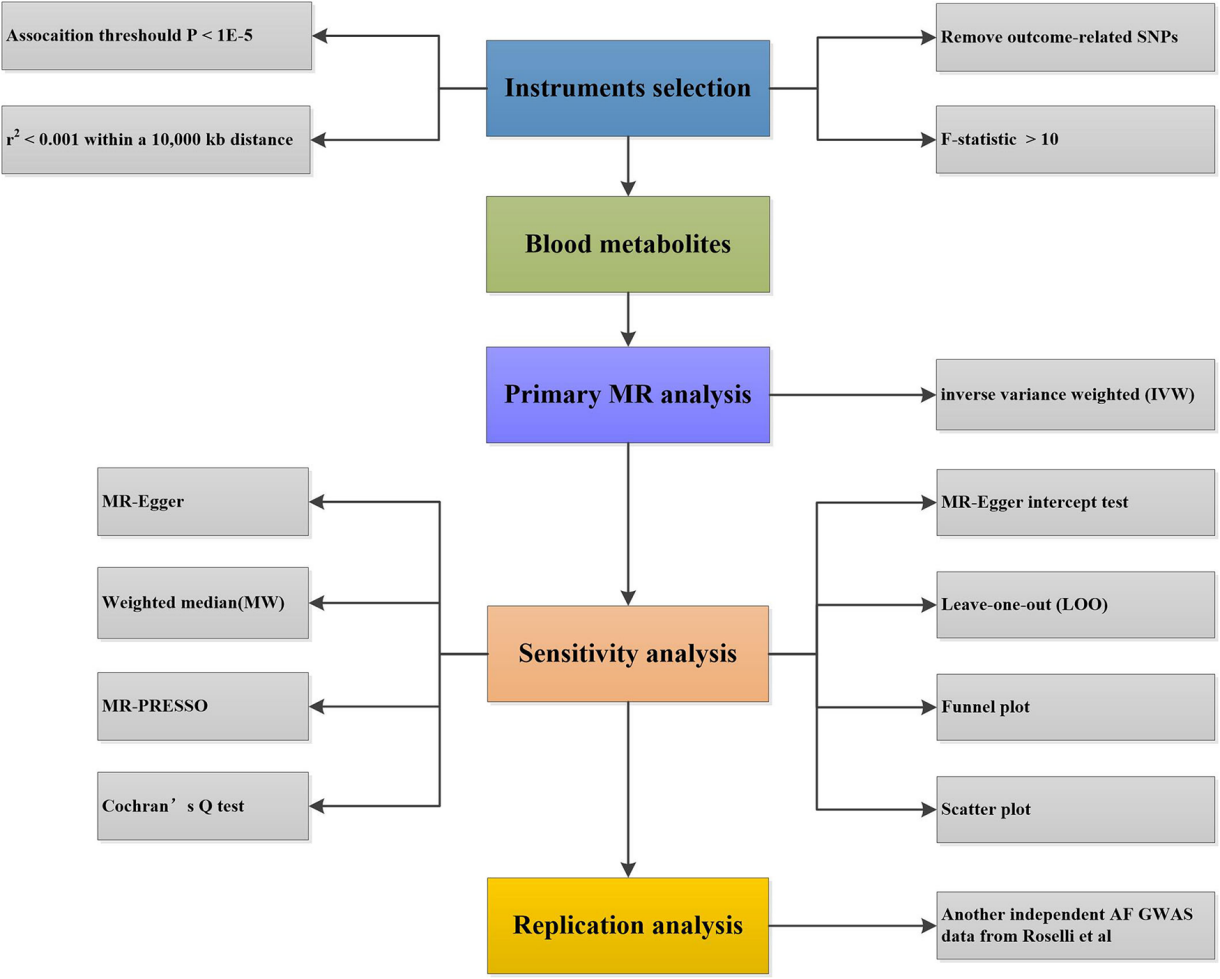
The flow chart of MR study revealing the causal relationship between human blood metabolites and the risk of AF. MR, Mendelian randomization; AF, atrial fibrillation; SNPs, single nucleotide polymorphisms; GWAS, genome-wide association study.

The study systematically evaluated the potential causal association between human blood metabolites and AF using the two-sample MR analysis method. The flow chart of the study is shown in Figure 1.

In essence, IVs must meet the following three assumptions: (1) IVs should be strongly correlated with the exposure; (2) IVs should be unrelated to any potential confounders; (3) IVs should be correlated with the outcome simply through exposure (17).

### 2.2. GWAS data for human blood metabolites

The GWAS data for human blood metabolites was from the most comprehensive genome-wide association estimation of blood metabolite so far conducted by Shin et al. (18). The study included 7,824 adults in Europe, including 1,768 from Germany and 6,056 from the United Kingdom. Through GWAS of non-targeted metabolomics, the correlation analysis of about 2.1 million SNPs in the population was carried out, and finally 486 blood metabolites were successfully obtained. Among them, there were 177 unknown metabolites and 309 known metabolites, which were further grouped into 8 groups (carbohydrates, lipids, amino acids, nucleotides, energy products, cofactors and vitamins, peptides and xenobiotics). Each participant in this study signed the informed consent form. Gene information of human blood metabolites was from Metabolomics GWAS server (https://metabolomics.helmholtz-muenchen.de/gwas/).

### 2.3. GWAS data for atrial fibrillation

The summary statistical data of GWAS for AF was from the largest GWAS study of AF by Nielsen et al. (19), with a total of 1,030,836 adults, including 60,620 patients with AF and 970,216 control subjects.

To further validate the results of the study, another AF GWAS data (from Roselli et al.) was repeatedly analyzed with MR method (20), which included 537,409 adults (55,114 patients with AF and 48,295 healthy controls).

### 2.4. Selection of instrumental variables

A series of steps were performed to screen out IVs related to blood metabolites. Firstly, given the finite amounts of SNPs with significant genome-wide effects, *P* < 1 × 10^−5^ was used as the threshold to extract IVs from 486 metabolites, which was consistent with previous studies (21–23). The European (EUR) 1000 Genomes Project Phase 3 reference panel was used to conduct linkage disequilibrium (LD) analysis to obtain independent IVs (the physical distance is within 10,000 kb; Linkage disequilibrium parameter *r*^2^ < 0.001). Also, in order to prevent deviation by using weak IVs, the F statistic of each metabolite was calculated to analyze the power of the IVs. To make sure that all SNPs had enough variation for the associated metabolite, weak IVs (those with *F* < 10) would be eliminated (24). Moreover, SNPs associated with the exposure were extracted from the outcome data and SNPs that were significantly (*P* < 1 × 10^−5^) related to the outcome were eliminated. Following that, harmonization was done to bring SNP-exposure and SNP-outcome alleles into alignment, and palindrome SNPs with moderate effective allele frequency and SNPs containing incompatible alleles were removed.

### 2.5. MR analysis

The primary MR method used to determine the causal link between exposure and outcome is the inverse variance weighted (IVW) model (13). Based on the presumption that all genetic variations are valid IVs, IVW is a highly effective method of analysis. When genetic variants satisfy the three IVs assumptions and are not affected by pleiotropy, IVW offers a strong consistency estimate of causal impact on exposure and outcome.

The *P* value adjusted for multiple hypothesis testing using Bonferroni's correction was *P* < 1.03 × 10^−4^ (0.05/486), suggesting a significant causal relationship (25). Meanwhile, *P* < 0.05 was considered to be nominally significant, the selected metabolites might be used as indicative risk predictors of AF.

### 2.6. Sensitivity analysis

This study used multiple sensitivity analysis methods to determine whether there was potential pleiotropy. Firstly, MR-Egger (26), Weighted Median (27) and MR-PRESSO (28) were used as the supplements of MR methods. These methods evaluated causal relationships using different postulated models. The consistency of directions of different MR analysis models increased the credibility of causal inference. Secondly, Cochran's *Q* value was used to assess the heterogeneity. If the *P* value was less than 0.05, it was considered that there was heterogeneity (29). Also, MR-Egger intercept method (30) and MR-PRESSO global test were used to measure the horizontal pleiotropy. If *P* < 0.05, it was thought that the horizontal pleiotropy had an impact on IVW, suggesting that the study results were unreliable. Then, Leave-one-out analysis was conducted to assess if the significant results were caused by specific IVs (30). There were also funnel plots, and scatter plots as supplements of sensitivity assessment.

In addition, we used the mRnd method (https://shiny.cnsgenomics.com/mRnd/) to estimate the power of MR analysis, which required input parameters: proportion of cases in the intended study (*K*), total sample size (*N*), true odds ratio of the outcome variable per standard deviation of the exposure variable (OR), proportion of variance in exposure variable explained by SNPs (*R*^2^) (31).

All analyses were conducted using R (version 4.2.3) and the “TwosampleMR”, “Mendelianmization” and “MR-PRESSO” R packages.

### 2.7. Replication analysis

In order to further verify the robustness of candidate blood metabolites, the same MR analysis was also conducted on another GWAS data for AF (from Roselli et al.), and the overlapping metabolites of the two analysis results were considered to have a significant causal relationship with AF.

In summary, the robust causal relationship between blood metabolites and AF in this study needed to meet the following conditions: firstly, Bonferroni's corrected *P* value (*P* < 1.03 × 10^−4^) was used as the significance threshold; Secondly, the results of sensitivity analysis showed no heterogeneity or pleiotropy; Thirdly, the results were verified by replication analysis.

### 2.8. Metabolic pathway analysis

MetaboAnalyst 5.0 (https://www.metaboanalyst.ca/) was used to analyze the selected blood metabolites to explore the relationship between metabolic pathways and AF. The dataset for pathway analysis came from the Small Molecular Pathway Database (SMPDB) and the Kyoto Encyclopedia of Genes and Genomes (KEGG).

## 3. Results

### 3.1. Strength of the instrumental variables

The number of SNPs of IVs generated by 486 blood metabolites ranged from 3 to 172. All F statistics of IVs were greater than 10 (the minimum *F* statistic was 17.64), indicating that IVs of 486 metabolites were effective enough for MR Analysis (Additional file 1: Supplementary Table S1). The harmonized data was shown in Additional file 1: Supplementary Table S1.

### 3.2. Effects of genetically determined metabolites on atrial fibrillation

Firstly, A total of 24 metabolites related to AF were analyzed by IVW method (*P* < 0.05), which included 6 types (1 carbohydrate, 5 lipids, 4 amino acids, 1 nucleotide, 3 xenobiotics and 10 unknown metabolites).

**Figure 2:**
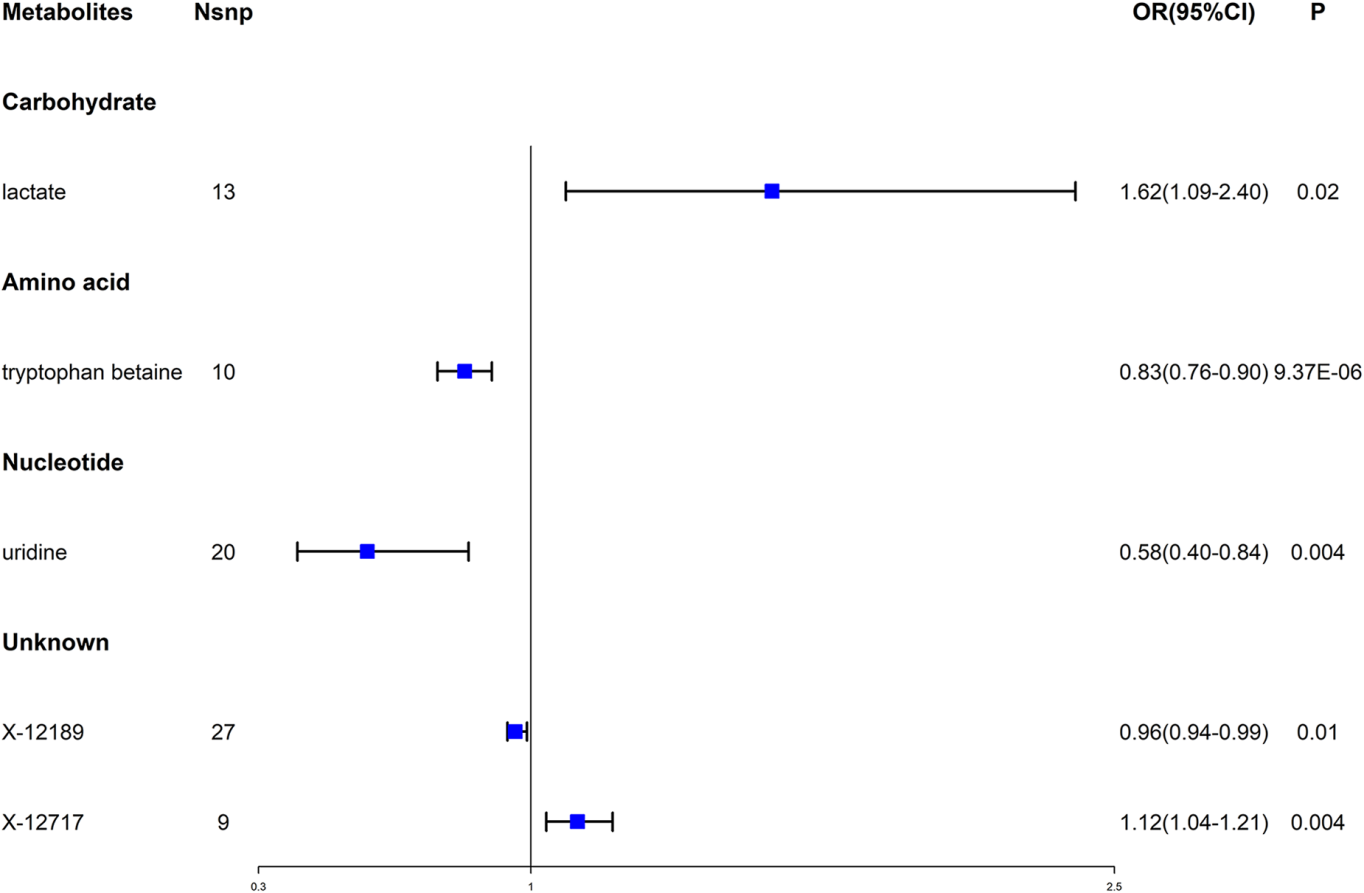
Forest plot for the causal effect of blood metabolites on the risk of AF. AF, atrial fibrillation; OR, odds ratio; CI, confidence interval.

Then, we used multiple sensitivity analysis methods to evaluate heterogeneity and pleiotropy (see Additional file 1: Supplementary Table S2 and Additional file 2: Supplementary Figure S1). Under strict screening conditions, 5 metabolites were finally selected, including 1 carbohydrate, 1 amino acid, 1 nucleotide and 2 unknown metabolites. Tryptophan betaine had a causal relationship with the reduced risk of AF (OR = 0.83, 95% CI = 0.76–0.90, *P* = 9.37 × 10^−6^), and the result met the adjusted threshold after Bonferroni's correction, which was the most statistically significant. While the remaining 4 metabolites had nominally significant causality with AF (*P* < 0.05), in which, uridine might have a causal relationship with the reduced risk of AF (OR = 0.58, 95% CI = 0.40–0.84, *P* = 0.004), Lactate might have a causal relationship with the increased risk of AF (OR = 1.62, 95% CI = 1.09–2.40, *P* = 0.02). X-12189 and X-12717 were 2 unknown metabolites, which might be associated with the risk of AF (see Figure 2).

### 3.3. Replication analysis

**Figure 3:**
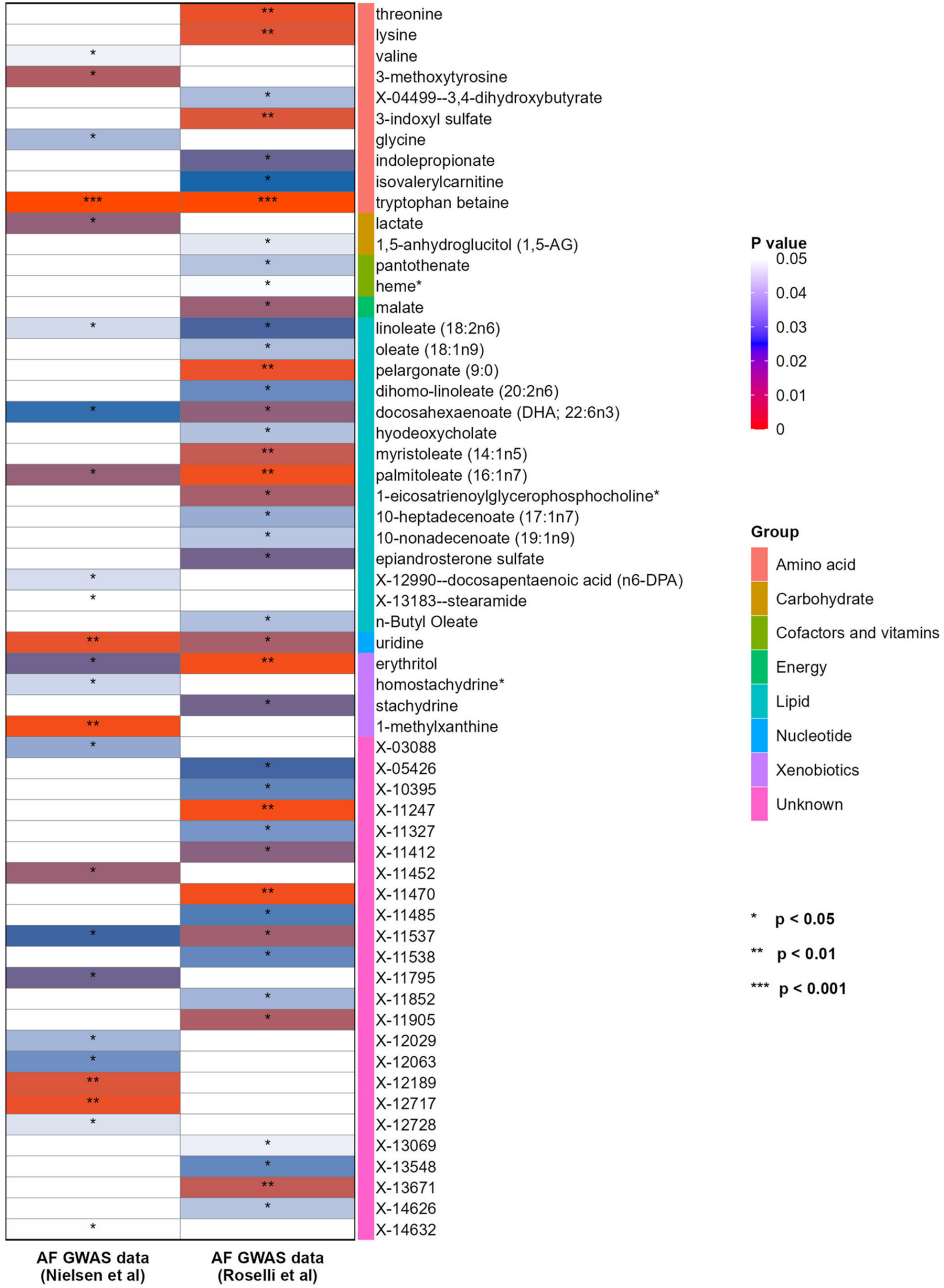
Heatmap showing the causal effect of blood metabolites on the two different GWAS datasets of AF (from IVW analysis). AF, atrial fibrillation; GWAS, genome-wide association study; IVW, inverse-variance weighted.

In order to further verify the results, another GWAS data for AF from Roselli et al. was repeatedly analyzed with the same method. Firstly, a total of 42 blood metabolites were selected by IVW method, including 13 lipids, 7 amino acids, 1 carbohydrate, 1 energy product, 2 cofactors and vitamins, 1 nucleotide, 2 xenobiotic and 15 unknown metabolites (see Figure 3).

After multiple sensitivity analyses, 8 blood metabolites were finally screened. Among them, 3 metabolites were associated with the increased risk of AF, including threonine (OR = 1.66, 95% CI = 1.18–2.34, *P* = 0.004), palmitoleate (16:1n7) (OR = 1.63, 95% CI = 1.19–2.24, *P* = 0.002) and X-11470 (OR = 1.51, 95% CI = 1.16–1.95, *P* = 0.002). 5 metabolites were associated with the reduced risk of AF, including tryptophan betaine (OR = 0.82, 95% CI = 0.76–0.88, *P* = 2.00 × 10^−7^), uridine (OR = 0.56, 95% CI = 0.35–0.88, *P* = 0.01), lysine (OR = 0.51, 95% CI = 0.32–0.82, *P* = 0.005), erythritol (OR = 0.75, 95% CI = 0.63–0.9, *P* = 0.002) and X-11247 (OR = 0.83, 95% CI = 0.74–0.93, *P* = 0.002).

**Figure 4:**
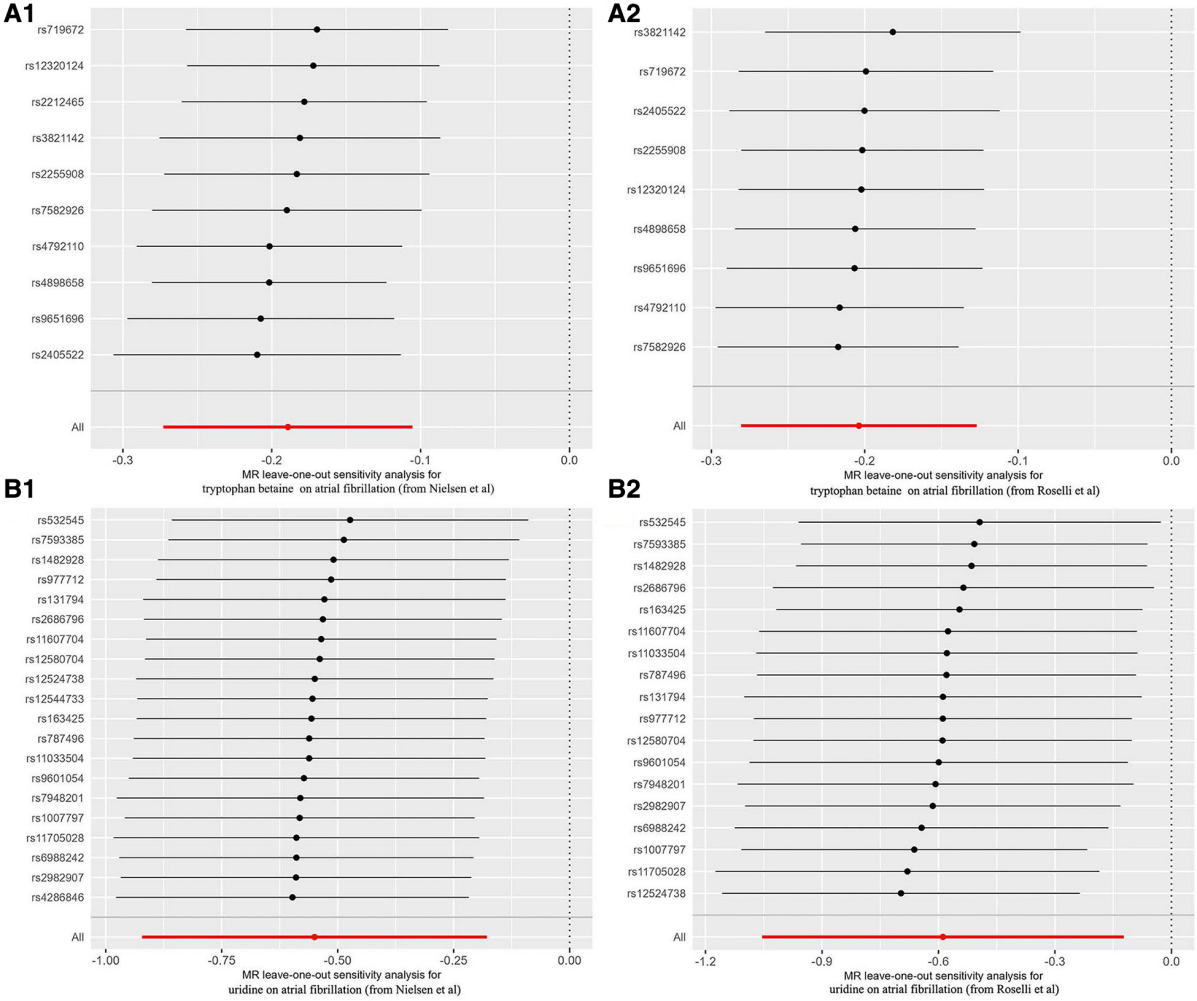
Forest plots for leave-one-out analysis of overlapped metabolite on atrial fibrillation. (**A1**) Tryptophan betaine on AF (from Nielsen et al); (**A2**) tryptophan betaine on AF (from Roselli et al); (**B1**) uridine on AF (from Nielsen et al); (**B2**) uridine on AF (from Roselli et al.). AF, atrial fibrillation.

Compared with the results of previous MR analysis on AF GWAS data (Nielsen et al.), it was found that there were 2 overlapped blood metabolites: tryptophan betaine and uridine. The causal relationship between tryptophan betaine and AF was the most significant (*P* < 1.03 × 10^−4^), while uridine had a nominally significant effect on AF (*P* < 0.05). The results of sensitivity analysis showed that none of them had obvious heterogeneity or pleiotropy (Table 1, Figure 4). And the statistical power of the above 2 metabolites on AF was 100%, thus confirming a robust cause-and-effect relationship.

**Table 1:** Sensitivity analysis results of overlapped metabolites on atrial fibrillation.

Metabolites	MR-Egger		Weighted median		MR-PRESSO		MR-Egger intercept	MR-PRESSO global test	Cochran's *Q* test
	OR (95% CI)	*P*	OR (95% CI)	*P*	OR (95% CI)	*P*	*P*	*P*	*P*
Tryptophan betaine									
AF (Nielsen et al.)	0.81 (0.64–1.02)	0.12	0.87 (0.79–0.96)	0.01	0.83 (0.76–0.90)	0.002	0.86	0.18	0.10
AF (Roselli et al.)	0.78 (0.63–0.95)	0.045	0.81 (0.72–0.90)	8.19 × 10^−5^	0.82 (0.77–0.87)	2.02 × 10^−4^	0.62	0.82	0.73
Uridine									
AF (Nielsen et al.)	0.49 (0.17–1.44)	0.21	0.70 (0.42–1.18)	0.18	0.58 (0.42–0.79)	0.003	0.75	0.82	0.80
AF (Roselli et al.)	0.84 (0.21–3.35)	0.81	0.57 (0.31–1.07)	0.08	0.56 (0.35–0.88)	0.02	0.53	0.35	0.32

OR, odds ratio; CI, confidence interval; *P*, *P*-value; AF, atrial fibrillation; GWAS, genome-wide association study.

### 3.4. Metabolic pathway analysis

Metabolite pathway analysis of blood metabolites derived from IVW was performed and identified three metabolic pathways related to AF (*P* < 0.05), including “Aminoacyl-tRNA biosynthesis” (*P* = 0.013), “Valine, leucine and isoleucine biosynthesis” (*P* = 0.031) and “Caffeine metabolism” (*P* = 0.038).

## 4. Discussion

Among 486 blood metabolites, we first identified 5 metabolites that had potential causal relationships with AF, including 3 known metabolites (tryptophan betaine, uridine, lactate) and 2 unknown metabolites (X-12189, X-12717). Then we performed the replication analysis on another GWAS dataset of AF to verify the results, and finally found 2 overlapping metabolites, among which tryptophan betaine was statistically significant and uridine was nominally significant.

AF is a common, complex and age-related arrhythmia that occurs in almost all organic and non-organic heart diseases. The high incidence and recurrence rate of AF have caused a great burden on people, so early diagnosis and prevention of AF are very important. Although many studies have explored the pathogenesis and pathological mechanisms of AF, the specific mechanism is not yet fully understood due to its complexity.

Tryptophan betaine, also known as lenticin or hypaphrine, is essentially an indole alkaloid, which is the N-methylated form of tryptophan. As an important active ingredient, it has played a very good role in anti-inflammatory, especially in endothelial cell injury. Sun et al. found that tryptophan betaine could inhibit endothelial cell inflammation induced by lipopolysaccharide (32–34). Ding et al. found that tryptophan betaine inactivated p38/JNK signal pathway by up-regulating DUSP1, thus preventing lipopolysaccharide-mediated inflammatory response (35). In addition, some studies found that tryptophan betaine analogues could prevent the changes of blood potassium and sodium ion concentrations during coronary artery occlusion/reperfusion injury in myocardial ischemia rats, and could effectively reduce myocardial reperfusion injury and infarction area (36). AF is the result of multiple mechanisms, including oxidative stress, autonomic nervous response, inflammation response and so on, and inflammation response is one of the key factors leading to the pathogenesis of AF (37, 38). Our results suggested that tryptophan betaine was negatively associated with the risk of AF. However, there were few previous studies on the correlation between tryptophan betaine and AF. According to previous research results about tryptophan betaine, it is speculated that tryptophan betaine may reduce the risk of AF through anti-inflammatory mechanism, but the exact biological mechanism needs to be clarified by further experimental studies.

Uridine, composed of uracil and D-ribose, is a component of RNA and belongs to pyrimidine nucleosides. As a necessary nucleoside, uridine exists in many biological fluids. Uridine is involved in some metabolic disorders, such as dhydropyrimidinase deficiency, mitochondrial neurogastrointestinal encephalopathy, beta-ureidopropionase deficiency and Lech-Nyhan syndrome, etc. Some studies found that uridine had protective effects on cardiovascular diseases. Early studies by Krylova et al. found that uridine and uridine-5′-monophosphate could reduce lactic acid, improve energy metabolism, and playe a protective role in myocardial ischemia and arrhythmias (including ventricular premature, ventricular tachycardia and ventricular fibrillation) (39, 40). Recent studies found that uridine could prevent myocardial injury in ischemia and reperfusion rat models by activating mitochondrial pathway, prevented the decrease of cell energy supply and antioxidant system activity, thus improving energy supply (41). Our study found that uridine may be negatively associated with AF, which is consistent with previous findings indicating the cardioprotective effect of uridine. Myocardial ischemia is an important cause of AF (42), and mitochondrial dysfunction is an important pathogenesis of AF (43), so uridine may improve mitochondrial function by regulating energy metabolism, thus improving myocardial ischemia to reduce the risk of AF. Nevertheless, it is still lack of sufficient experimental evidence.

Lactate was found to have a possible causal relationship with AF in the initial MR analysis. Although it was not finally verified in the replication analysis, its relationship with AF is still worth exploring. Lactate is an organic acid that is a chiral molecule composed of two optical isomers, L-lactic acid and D-lactic acid. Lactate is continuously produced from pyruvate through lactate dehydrogenase (LDH) during normal metabolism and exercise fermentation. Plasma lactate is the final product of anaerobic metabolism of glucose and a sign of the imbalance between oxygen supply and demand in the body. Previous studies (44, 45) showed that plasma lactate was closely associated with AF, which played a potential role in the maintenance and recurrence of AF. When the plasma lactate increases excessively, the heart is in a state of hypoxia and the energy supply of myocardial cells is insufficient. This can stimulate the production of reactive oxygen species, lead to oxidative stress and mitochondrial controlled apoptosis, cause myocardial structural remodeling (46), and promote the occurrence and development of AF. In our study, lactate was positively correlated with AF, which was consistent with the above studies.

**Figure 5:**
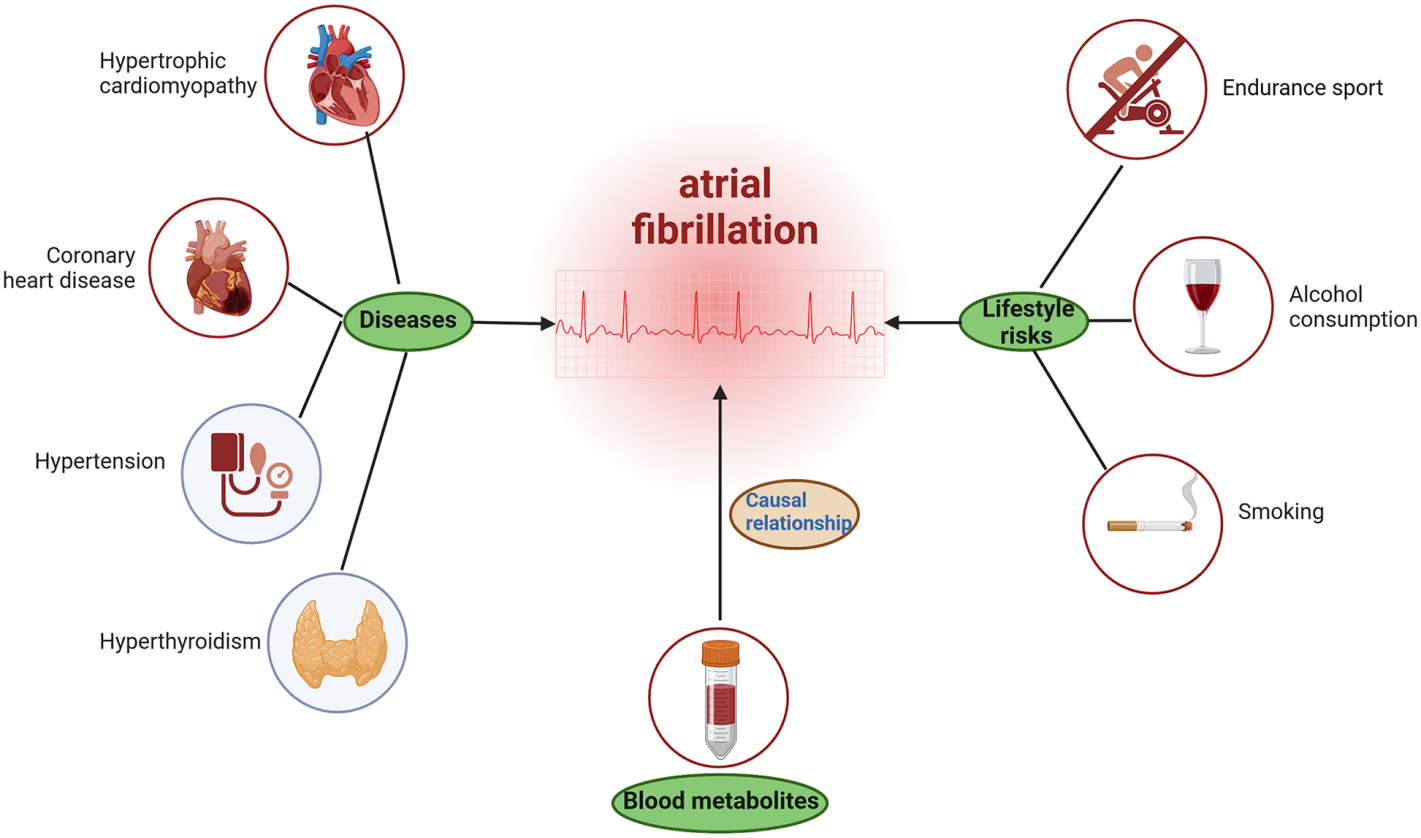
Possible potentially causes of atrial fibrillation. The figure was created with BioRender.com.

The mechanisms of AF may be a combination of multiple factors. This study found a causal relationship between some blood metabolites and AF, which helps us understand the pathogenesis of AF. However, we cannot ignore other risk factors related to AF. For example, in the Brugada syndrome, the underlying mechanism of AF may include genetic or acquired factors that may affect autonomic nervous function, atrial structure, conduction velocity, etc. (47). In the hypertrophic cardiomyopathy, AF is linked to advanced diastolic dysfunction, left atrial dilation, and remodeling (48). Moreover, AF may be precipitated by hypertension, hyperthyroidism (49), lifestyle factors such as endurance sport (50), alcohol consumption, and even smoking (51) (see Figure 5).

Our study aims to investigate whether there is a causal relationship between blood metabolites and AF. Through relatively rigorous MR analysis, we obtained some blood metabolites that have a potential causal relationship with AF, and determined the possible metabolic pathways through metabolic pathway analysis. This is exploratory research and analysis, which lays a solid foundation for further targeted metabolomics research in the future.

The study had some strengths. First, this study covered a wide range of human blood metabolites (486 in total) as exposure factors for MR analysis, aiming to study the metabolic characteristics leading to AF. Second, MR method effectively overcame the defects of traditional observational studies, and avoided reverse causation and residual confounding to a large extent. Third, after MR analysis of two different GWAS datasets of AF, overlapping blood metabolites were screened out, which could better explain the causal effect of these metabolites on AF, providing more reliable evidence. Fourth, the study used Bonferroni's corrected *P* value due to multiple testing, thus the results were more scientific and rigorous.

However, the study had several limitations. First, because the number of SNPs meeting genome-wide effect was limited, the *P* value was relaxed, but the F statistic of all SNPS was greater than 10 to exclude weak IVs, which is a commonly used strategy. Second, given the classification of the original datasets, it was impossible to further subdivide the clinical types of AF, so only an overall analysis could be performed. Third, all of the GWAS databases in this article are from Europe, so it is not clear whether the same results would be produced and extended to other ethnic groups. Further research is needed in the future to evaluate its generality in other ethnic groups. Fourth, although MR method has been proven to be an effective tool for assessing the causality between the exposure and the outcome, the findings need to be further studied depending on some experimental observations to reveal their significance in the development of AF.

## 5. Conclusion

In summary, this study used the two-sample MR analysis to analyze the effects of 486 blood metabolites on the risk of AF. The results indicated that there was a significant negative correlation between tryptophan betaine and the risk of AF. While uridine might have a potential causal relationship with AF. This study strengthens our understanding of the relationship between blood metabolites and AF, hoping to provide more useful information for future research on the pathogenesis of AF.

## Data Availability

The original contributions presented in the study are included in the article/**Supplementary Material**, further inquiries can be directed to the corresponding author.
