# Supplementary material for: The causal effects of genetically determined human blood metabolites on the risk of atrial fibrillation
Source: Front Cardiovasc Med. 2023 Jul 26;10:1211458. doi: 10.3389/fcvm.2023.1211458 (PMC10410273; doi:10.3389/fcvm.2023.1211458)
Supplement: Supplementary file 2 [file Table2.docx]

*Additional file 2*


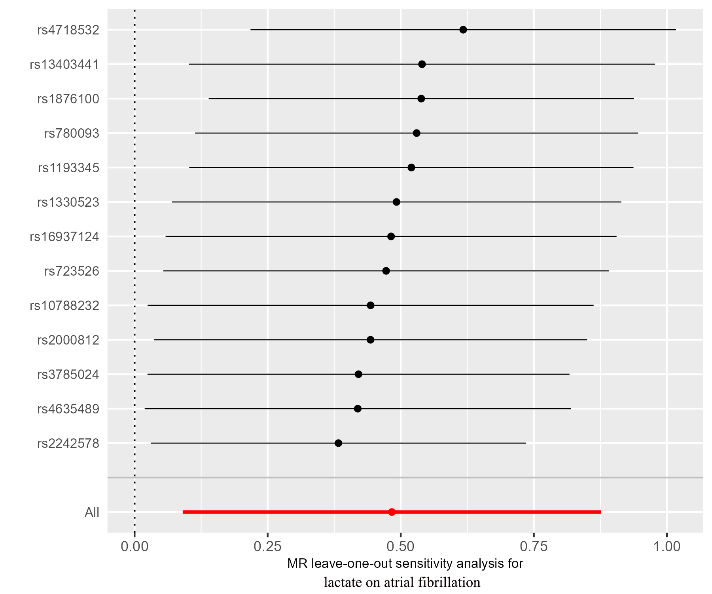

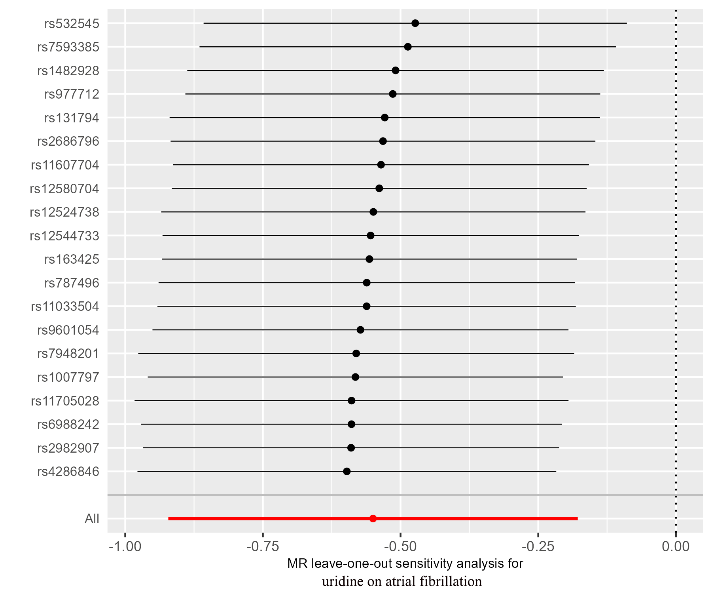

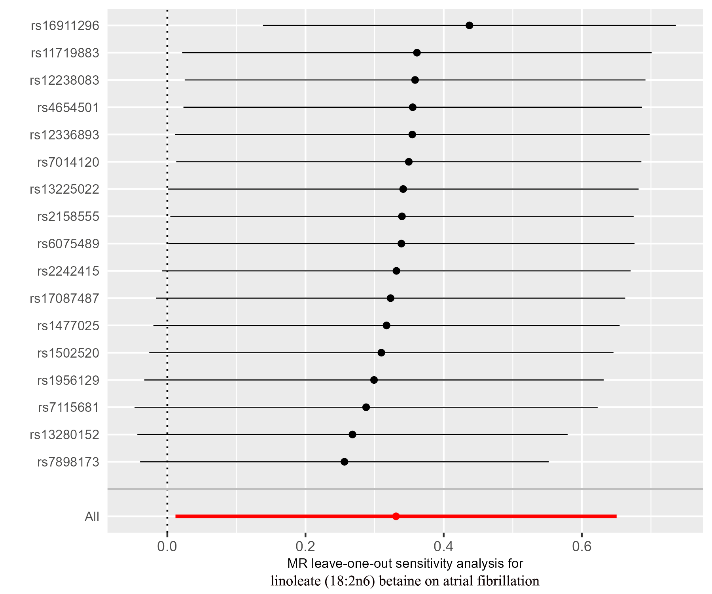

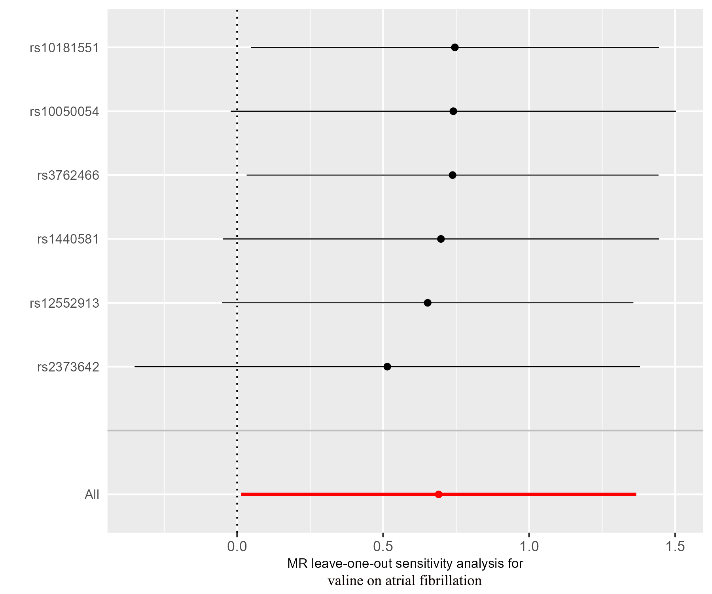

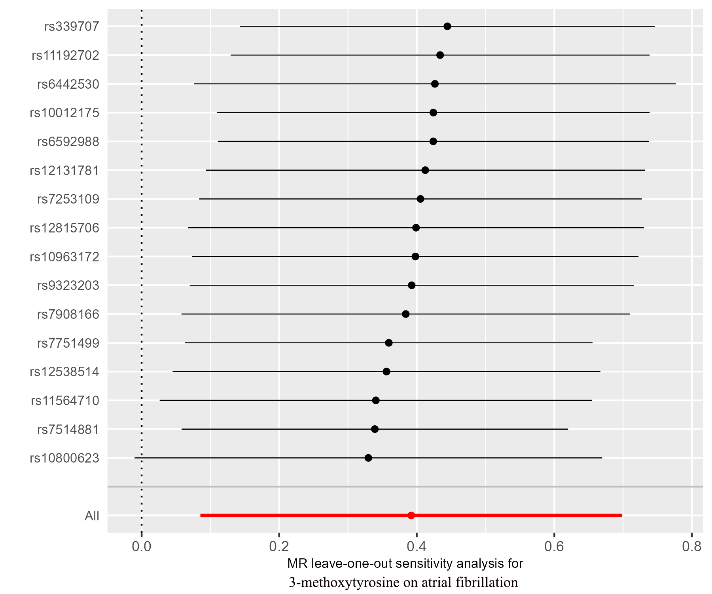

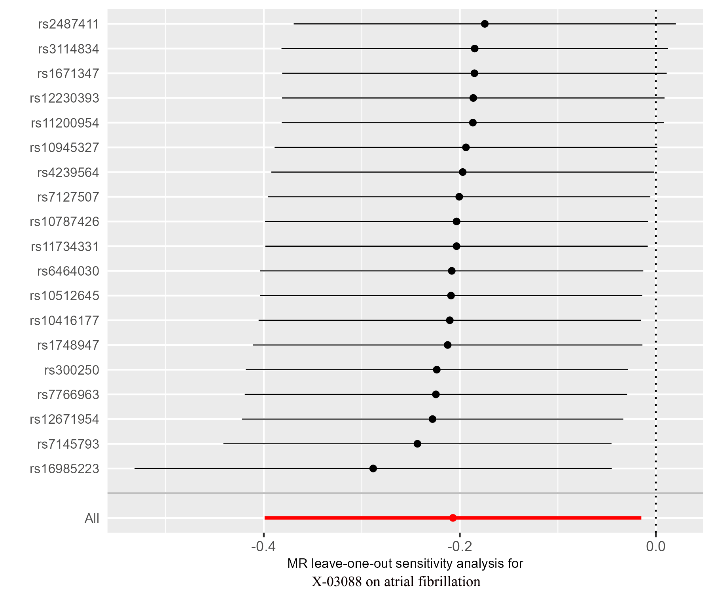

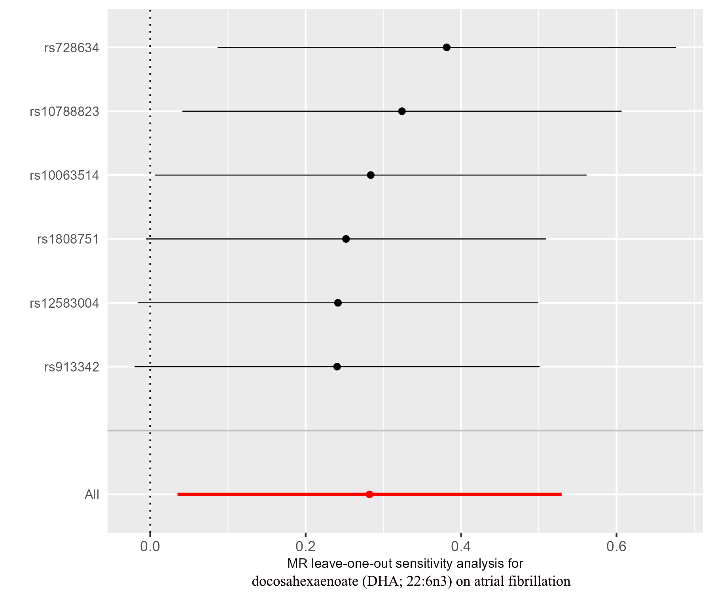

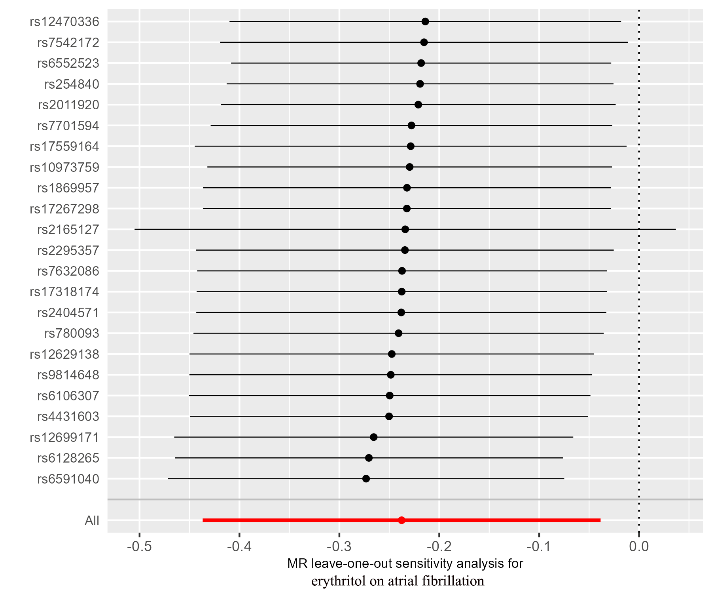

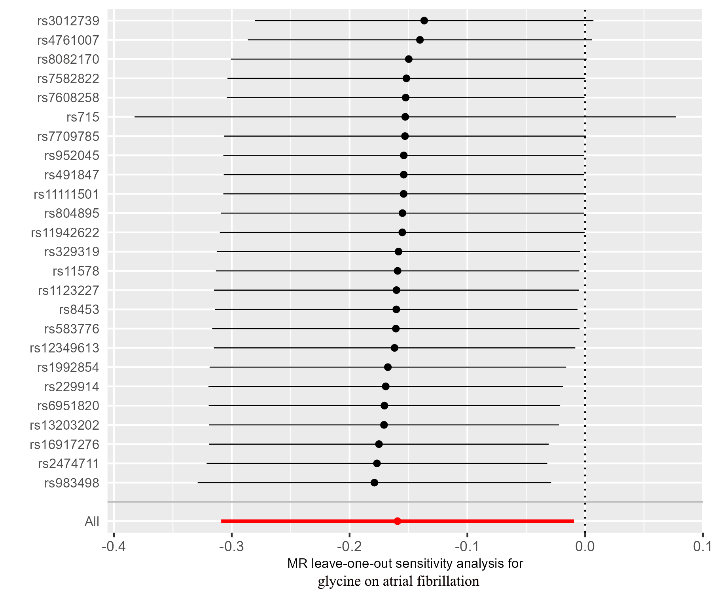

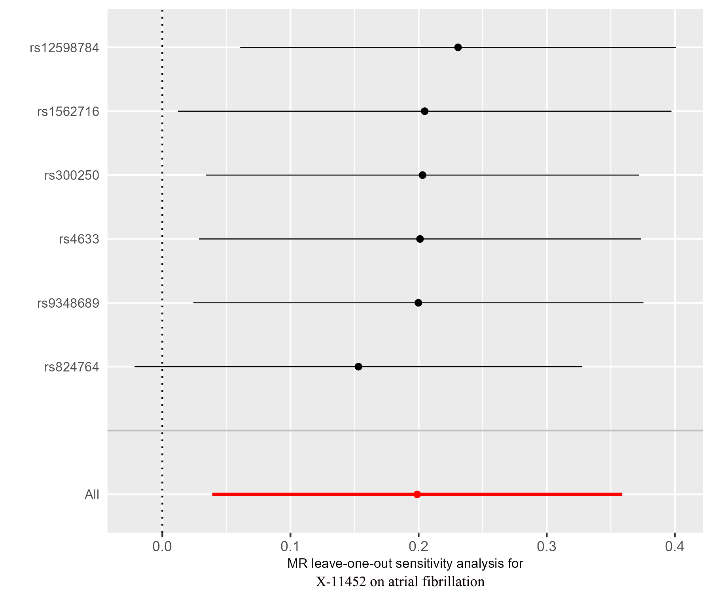

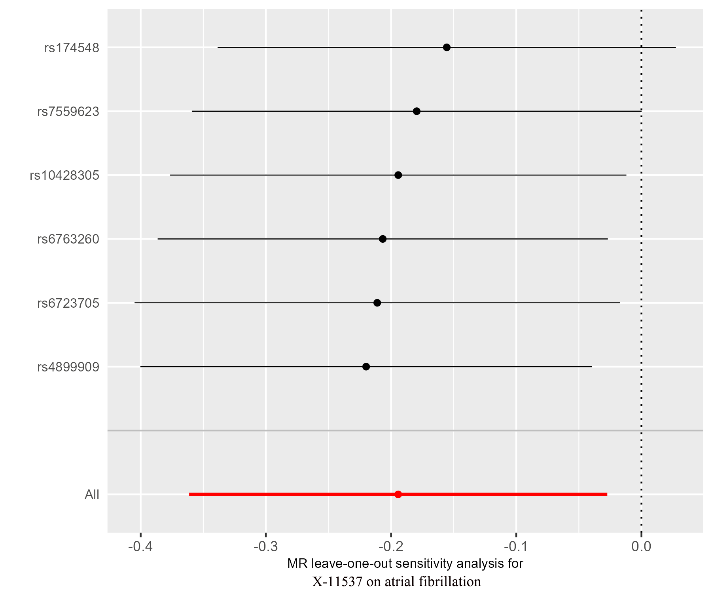

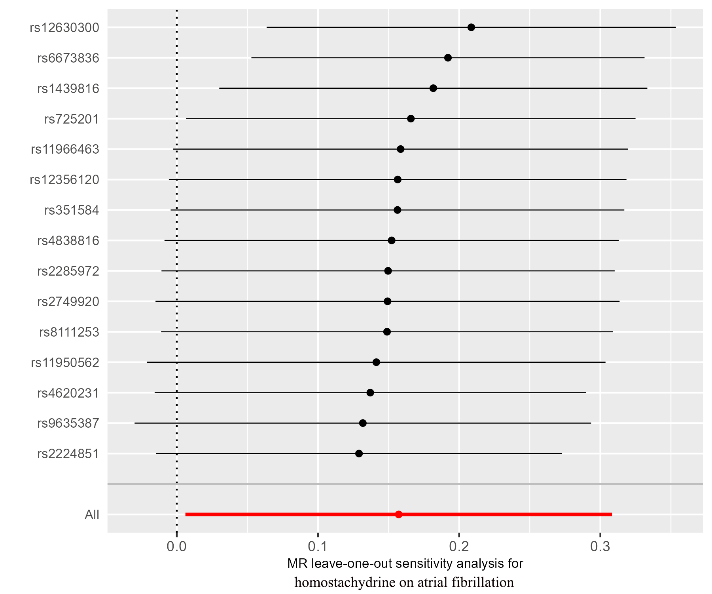

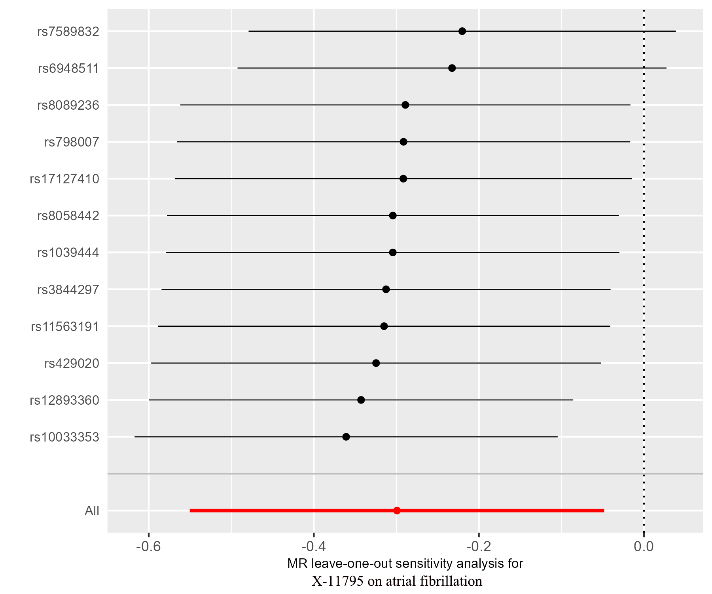

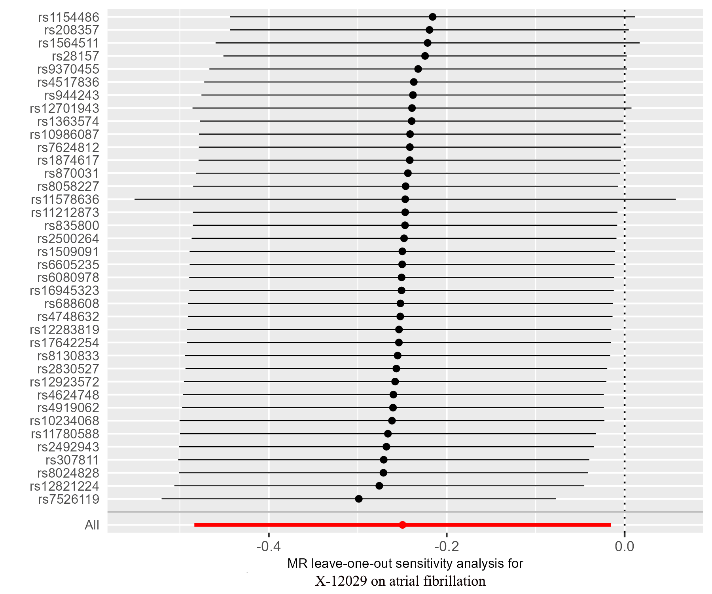

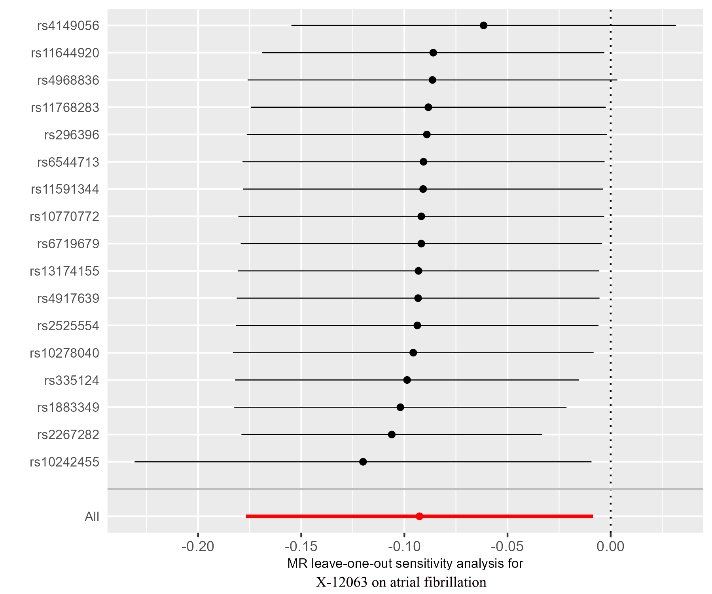

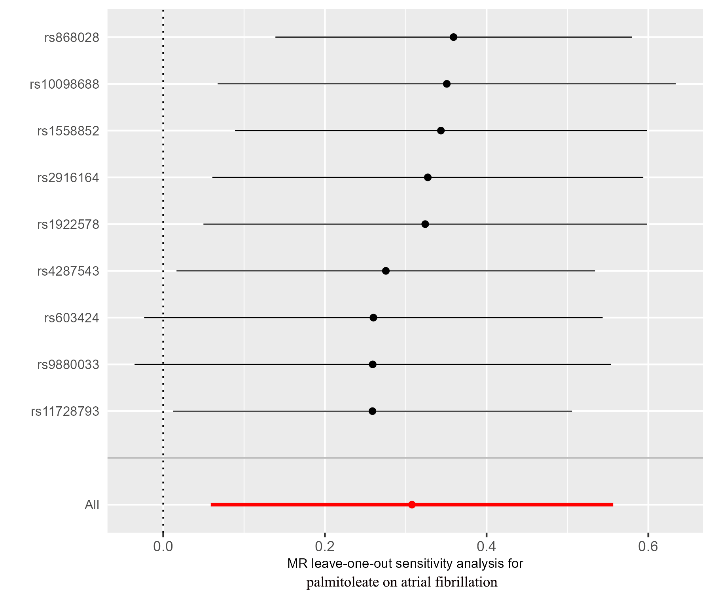

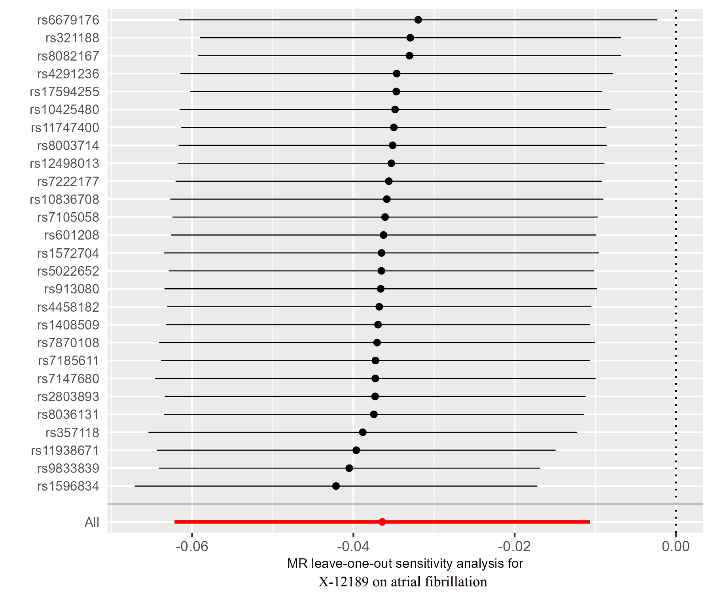

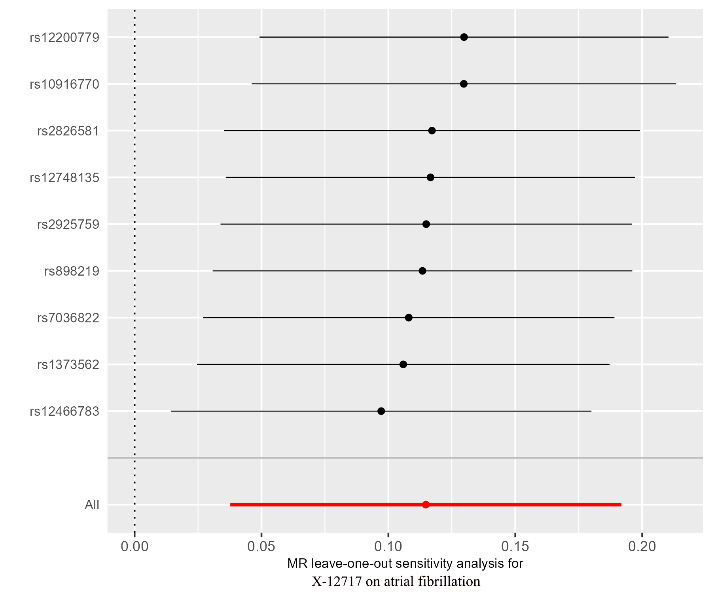

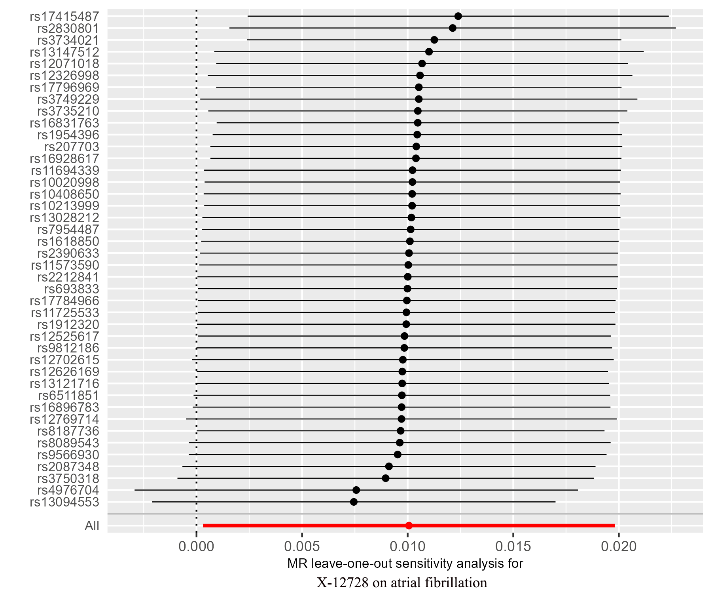

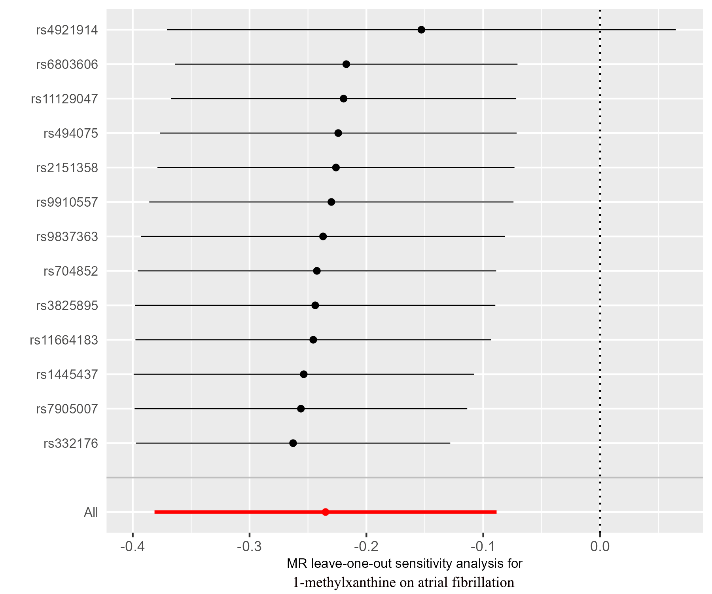

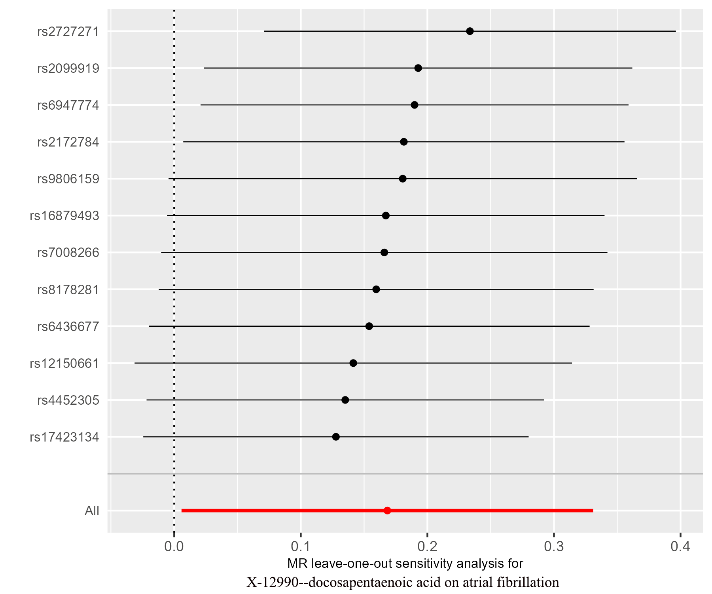

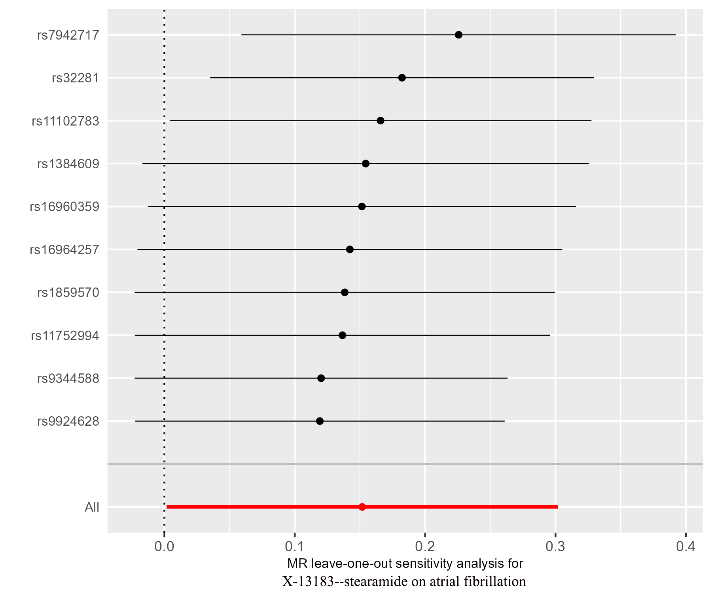

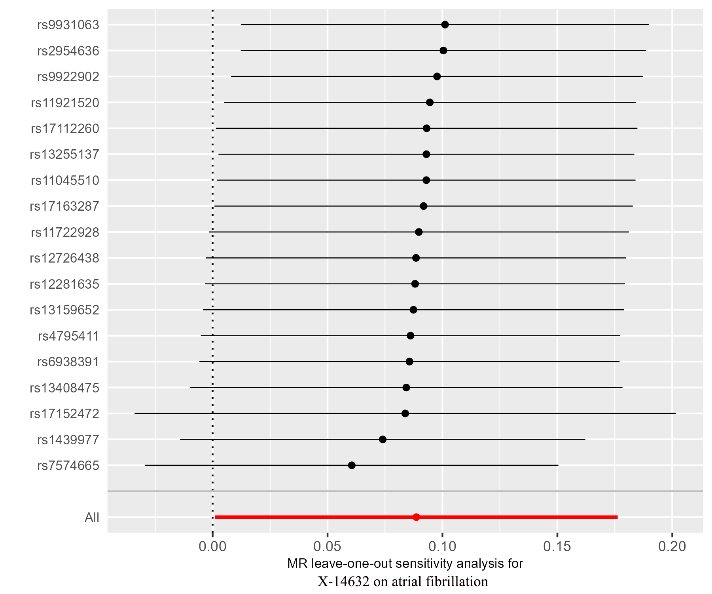

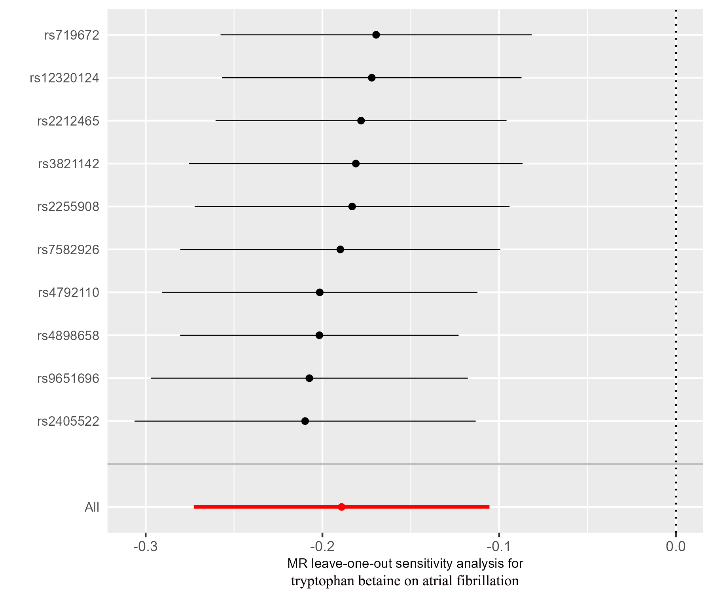


**Figure S1** Leave-one-out plots for blood metabolites on the risk of atrial fibrillation based on the inverse variance weighted (IVW) method
